# [COMMODE] a large-scale database of molecular descriptors using compounds from PubChem

**DOI:** 10.1186/1751-0473-8-22

**Published:** 2013-11-13

**Authors:** Andreas Dander, Laurin AJ Mueller, Ralf Gallasch, Stephan Pabinger, Frank Emmert-Streib, Armin Graber, Matthias Dehmer

**Affiliations:** 1 UMIT, Division for Bioinformatics and Translational Research, Eduard Wallnoefer Zentrum 1, A-6060 Hall in Tyrol, Austria; 2 Biocenter Innsbruck, Division for Bioinformatics, Innsbruck Medical University, Innrain 80-82, A-6020 Innsbruck, Austria; 3 Oncotyrol, Center for Personalized Medicine, Karl-Kapferer-Straße 5, A-6020 Innsbruck, Austria; 4 Computational Biology and Machine Learning Laboratory, Center for Cancer Research and Cell Biology, School of Medicine, Dentistry and Biomedical Sciences, Queen’s University Belfast, 97 Lisburn Road, Belfast BT9 7BL, UK

**Keywords:** Chemical databases, Molecular descriptors, PubChem, QSAR, QSPR

## Abstract

**Background:**

Molecular descriptors have been extensively used in the field of structure-oriented drug design and structural chemistry. They have been applied in QSPR and QSAR models to predict ADME-Tox properties, which specify essential features for drugs. Molecular descriptors capture chemical and structural information, but investigating their interpretation and meaning remains very challenging.

**Results:**

This paper introduces a large-scale database of molecular descriptors called COMMODE containing more than 25 million compounds originated from PubChem. About 2500 DRAGON-descriptors have been calculated for all compounds and integrated into this database, which is accessible through a web interface at http://commode.i-med.ac.at.

## Background

Molecular descriptors have been proven essential in drug design, structural and mathematical chemistry, bioinformatics, and related disciplines [1-7]. Descriptors can be understood as functions to map chemical or structural properties of chemicals to positive real numbers. The latter often results in so-called topological or structural descriptors [7,8] by deriving structural features of molecular structures. Following Todeschini et al. [7], we realize that a vast amount of molecular descriptors have been developed; surprisingly, many of their properties have not yet been properly explored. Table 1 lists the existing categories of molecular descriptors calculated using DRAGON [9].

**Table 1:** Groups of molecular descriptors

**Group**	**Name of the group**	**Number of descriptors**
B01	Constitutional descriptors	48
B02	Topological descriptors	119
B03	Walk and path counts	47
B04	Connectivity indices	33
B05	Information indices	47
B06	2D Autocorrelations	96
B07	Edge adjacency indices	107
B08	Burden eigenvalue descriptors	64
B09	Topological charge indices	21
B10	Eigenvalue-based indices	44
B17	Functional group counts	154
B18	Atom-centered fragments	120
B20	Molecular properties	29
B21	2D binary fingerprints	780
B22	2D frequency fingerprints	780

This table lists the different groups for all computed molecular descriptors created by DRAGON. In total 2,489 molecular descriptors were calculated and integrated into COMMODE.

The main contribution of this paper is to provide a large-scale, online available database, containing over 25 million chemicals downloaded from the database PubChem[10,11]. Our database, called COMMODE (COMpilation of MOlecular DEscriptors), provides a valuable source containing descriptor data, which is usually not available at a large scale. As we have already mentioned, the present paper can be regarded as an application explaining the database as well the underlying tool in brief. It would go far beyond the scope of the paper to explain the features and the application of COMMODE in in depth. COMMODE also allows researchers to examine the *interpretation* or *meaning*[5] of molecular descriptors.

In the context of topological descriptors [1,3,5,6], this relates to exploring the *structural interpretation* of such measures. Exemplarily, branching, cyclicity, connectivity and symmetry are plausible interpretations of such indices that have been investigated, see [5,12-15]. Nevertheless, one is far away from possessing a general framework to tackle this problem by using a large number of available descriptors. We believe that COMMODE will prove to be useful to resolve this challenging task successfully. Also, researchers who are active in QSPR and QSAR [16,17] and those developing chemometrics-driven models [18] using descriptor data might utilize this database.

The paper is organized as follows. In the section ‘Implementation’, we explain the database scheme, and details of the data generation and integration process. The section ‘Results and discussion’ outlines the usage of our tool with a standard web browser. The article finishes with ‘Conclusions’.

## Implementation

### Molecular and structural descriptors

Molecular descriptors encode certain information about chemicals. As a result, special classes of such measures have been developed to emphasize particular aspects of chemicals, e.g., atom types, bond types or structural properties. In particular, molecular descriptors have been proven essential for designing QSPR/QSAR models efficiently [16,17]. In this application, we calculated descriptors using of DRAGON [9]. A class of descriptors that has been investigated extensively are topological (or structural) descriptors [5-8]. Clearly, this class itself can be divided in different subcategories such as graph entropy [8,19] representing information-theoretic indices, eigenvalue-based measures [20,21], distance-based measures [7,22] and symmetry-based descriptors [7]. Note that DRAGON has its own categorization of molecular descriptors and, for instance, it does not consider information-theoretic descriptors representing the structural information content of a chemical structure as topological descriptors.

From a practical point of view, molecular descriptors have been used extensively to predict melting and boiling points [23]. Also, other chemical properties such as properties that are important in the drug design process have been used in combination with molecular descriptors. Crucial properties can be ADME-Tox properties (absorption, distribution, metabolism, excretion, and toxicity) influencing different essential aspects of drugs [24]. Examples for molecular descriptors infulencing ADME-Tox properties are the octanol/water partition coefficient (LogP) [25], the aqueous solubility description (LogS) [26] and the blood-brain barrier permeation (LogBB) [27].

### Large-scale database of molecular descriptors

This section describes the MySQL database scheme and the process of data integration with Java routines, as well as the calculation of the molecular descriptors by using DRAGON. Moreover, the web page, which provides access to the large scale database and is essential for querying the database, and the implemented descriptive analysis, is explained.

COMMODE contains about 25 million chemicals downloaded from the database PubChem and about 2,500 molecular descriptors, which have been calculated for all of those compounds using DRAGON [9]. The database can be accessed through a php-based web application at http://commode.i-med.ac.at[28], where different queries can be started, files with compounds of interest can be uploaded, and results can be exported. Furthermore, additional descriptive values can be calculated and statistically analyzed by means of statistical measures, correlation and uniqueness.

#### Compounds

As we used compounds from PubChem, the first step was to download the complete set provided as SDF-files [29] (Structure Data Format). Those files contain 25,051,770 compounds, which vary largely in their size and structure. The mass of the downloaded compounds ranges from 1 to 59750 Dalton with a median value of 383 Dalton, whereby 45 compounds are heavier than 10,000 Dalton. Also the number of heavy atoms (anything other than hydrogen) varies dramatically between 0 and 576 with a median at 27. Other constitutional information about the compounds integrated in COMMODE are number of atoms (min=1, max=982, median=48), number of bonds (min=0, max=990, median=50) and the number of rings (min=0, max=19, median=3) per molecule.

#### Integration of compounds

**Figure 1:**
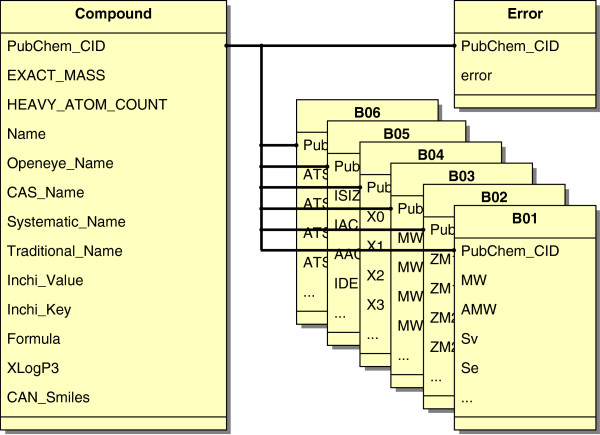
**Database scheme for compounds.** The main relation in COMMODE is the relation *Compound* including attributes loaded from PubChem. This relation is linked by using the PubChem identifier with one relation for each of the 15 groups of descriptors (B01-B10, B17, B18, B20-B22) calculated using DRAGON. Each group contains a variety of molecular descriptors (listed in Table 1). For simplification just the first six groups with their corresponding first four descriptors are shown here. Furthermore, the relation *Error* holds a Boolean value, which is true if a problem occurred using DRAGON for this molecule.

A tailored Java routine has been developed for the correct integration of general information about compounds into a MySQL database [30]. In general, this Java routine reads the data from the unpacked SDF-files and creates one entry per compound with attributes such as the PubChem identifier, exact mass, heavy atom count, SMILES [31] format, and the systematic name for all of the 25 million compounds. Figure 1 shows the database scheme for compounds including the central relation *Compound* containing attributes obtained from PubChem. A second Java routine creates one relation for each single DRAGON group, including molecular descriptors as attributes and the PubChem identifier. All of the mentioned relations are connected to *Compound* by using PubChem_CID, which is the primary key in those relations.

#### Computation and integration of molecular descriptors

After the successful completion of the compound integration, 2,489 molecular descriptors have been calculated by applying DRAGON to all SDF-files. Table 1 lists all 15 calculated groups, their name, and their corresponding number of descriptors. DRAGON produces one file per input file and per group, thereby, numerous files were created holding a huge number of positive real numbers. It appears that DRAGON was not able to calculate the descriptors for 950,688 compounds, due to different errors. These errors can have different causes, like there is just one atom (e.g. hydron H^+^ (id 1038), bromide Br^-^ (id 259)), or the downloaded molecule represents an unconnected graph and therefore those molecular descriptors can not be computed (e.g. [3-(dimethylcarbamoyloxy)phenyl]-trimethyl-ammonium; methyl sulfate (id 5824)). As DRAGON fails to calculate molecular descriptors for these compounds, the relation *Error* with the Boolean attribute error was introduced. As 3D information of the compounds is not available using the provided SDF-files, the corresponding 3D descriptors have not been calculated. To handle the large number of result files, an additional Java routine was developed, which integrates those files into the previously generated database relations, where the first attribute for each group contains the identifier from PubChem.

**Figure 2:**
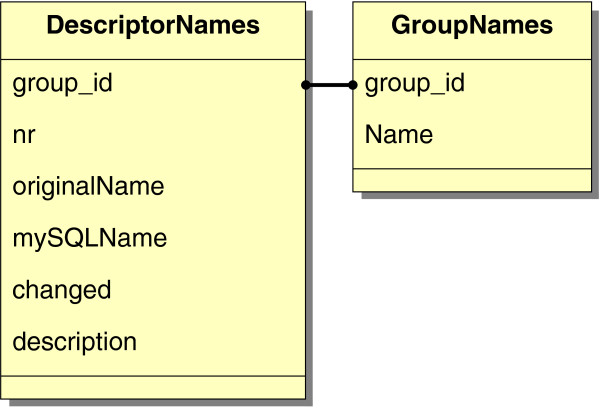
**Database scheme for meta data about descriptors and its groups.** This part of COMMODE contains general information about descriptors and their corresponding groups integrated in the relations *DescriptorNames* and *GroupNames*.

Meta-data about molecular descriptors and their corresponding groups can also be found in the database scheme. Therefore, the relation *DescriptorNames* contains attributes describing a molecular descriptor. *DescriptorNames* is associated with the relation *GroupNames*, which stores the name of all DRAGON groups. Figure 2 depict those two relations with their corresponding attributes.

## Results and discussion

### Querying COMMODE

**Figure 3:**
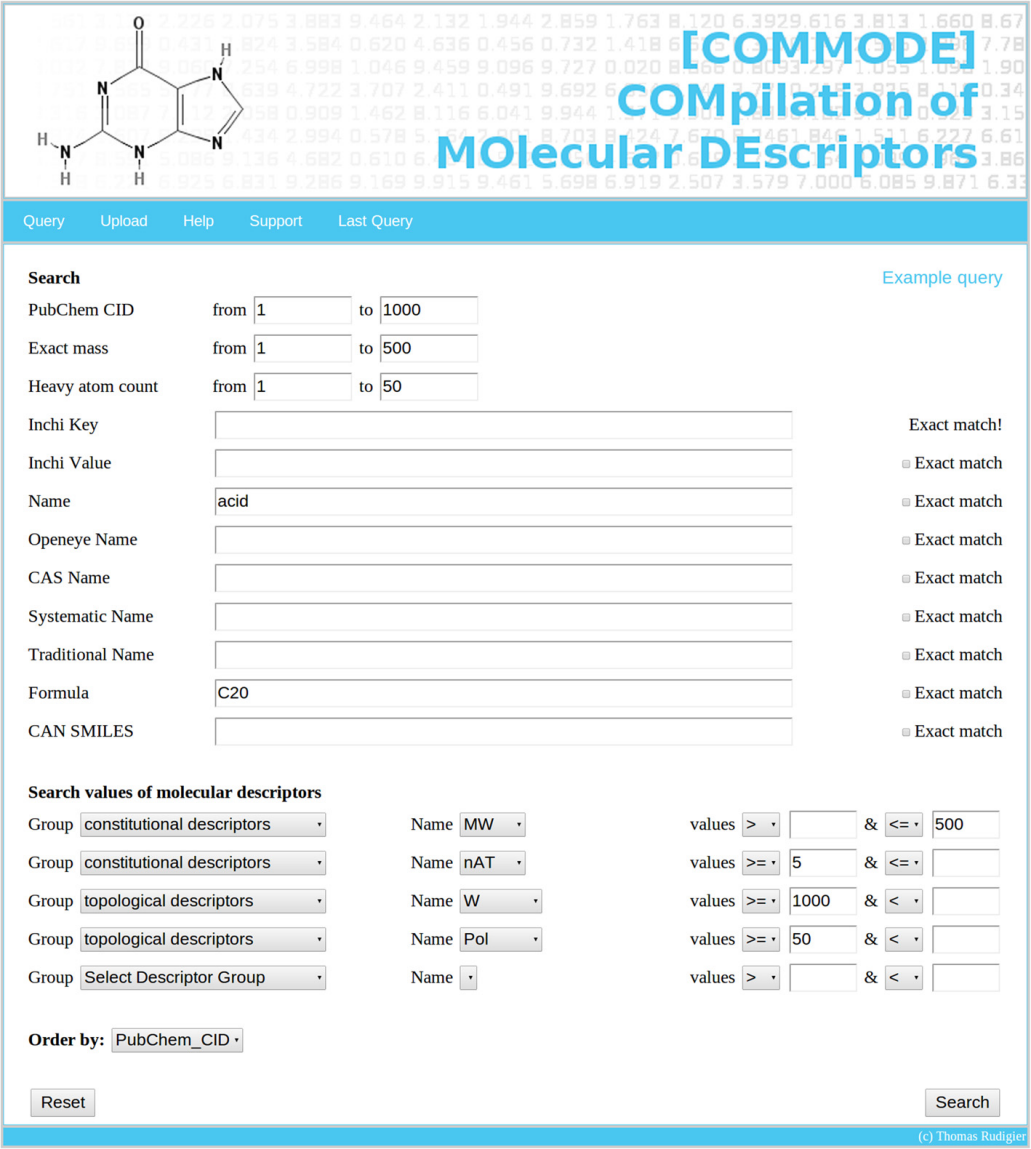
**Screen-shot of COMMODE showing the querying page.** This screen-shot shows the query page of COMMODE with all different search criteria like PubChem identifier, exact mass, systematic name, smiles, or value ranges for five different molecular descriptors. The results of the given query lists 6 compounds.

A php-based web application has been developed, which manages the access to the MySQL database. The user can search COMMODE in two different ways. The first way is based on providing different search criteria like the PubChem identifier, exact mass, heavy atom count, different types of name, molecular formula, and ranges for various molecular descriptors. All search criteria are concatenated using a Boolean “AND”, which means that all found molecules must fulfill any given search criteria. Figure 3 shows a screen-shot of the web page including attributes, which are provided for querying the database. The second more flexible way is to provide a CSV-file of interesting compounds using their corresponding PubChem identifier. COMMODE uses this list and is able search for corresponding values of all integrated molecular descriptors. As the database contains around 25 million compounds the resulting tables can be huge, for performance reasons we limit the query result to 1,000 tuples.

### Views on the data

After querying COMMODE various views displaying the results are provided. The first view shows a list with general information about the resulting compounds. Each single molecule can be selected from this list and explored. Therefore, a view was implemented showing different names and values for each molecule as well as a link to the corresponding PubChem page. This view also shows a 2D and a 3D plot from the molecule derived from PubChem. The user can further see the values of all molecular descriptors for the compound of interest or the values from a single molecular descriptor for all compounds of the given query.

### Statistical analysis

When analyzing data of molecular descriptors on a large-scale, a statistical analysis is crucial. For example, this relates to estimating the correlations between descriptors to examine whether they capture chemical or structural information of compounds similarly. Note that this problem has been already tackled by Basak et al. [12] and Todeschini et al. [32]; but we would like to emphasize that in their analysis they have only used small subsets of compounds and descriptors. COMMODE offers now the opportunity to investigate this problem on a large-scale without the need to having access to a stand-alone application that computes molecular descriptors. This might be particularly interesting for researchers who want to analyze properties of molecular descriptors by using existing compounds.

**Figure 4:**
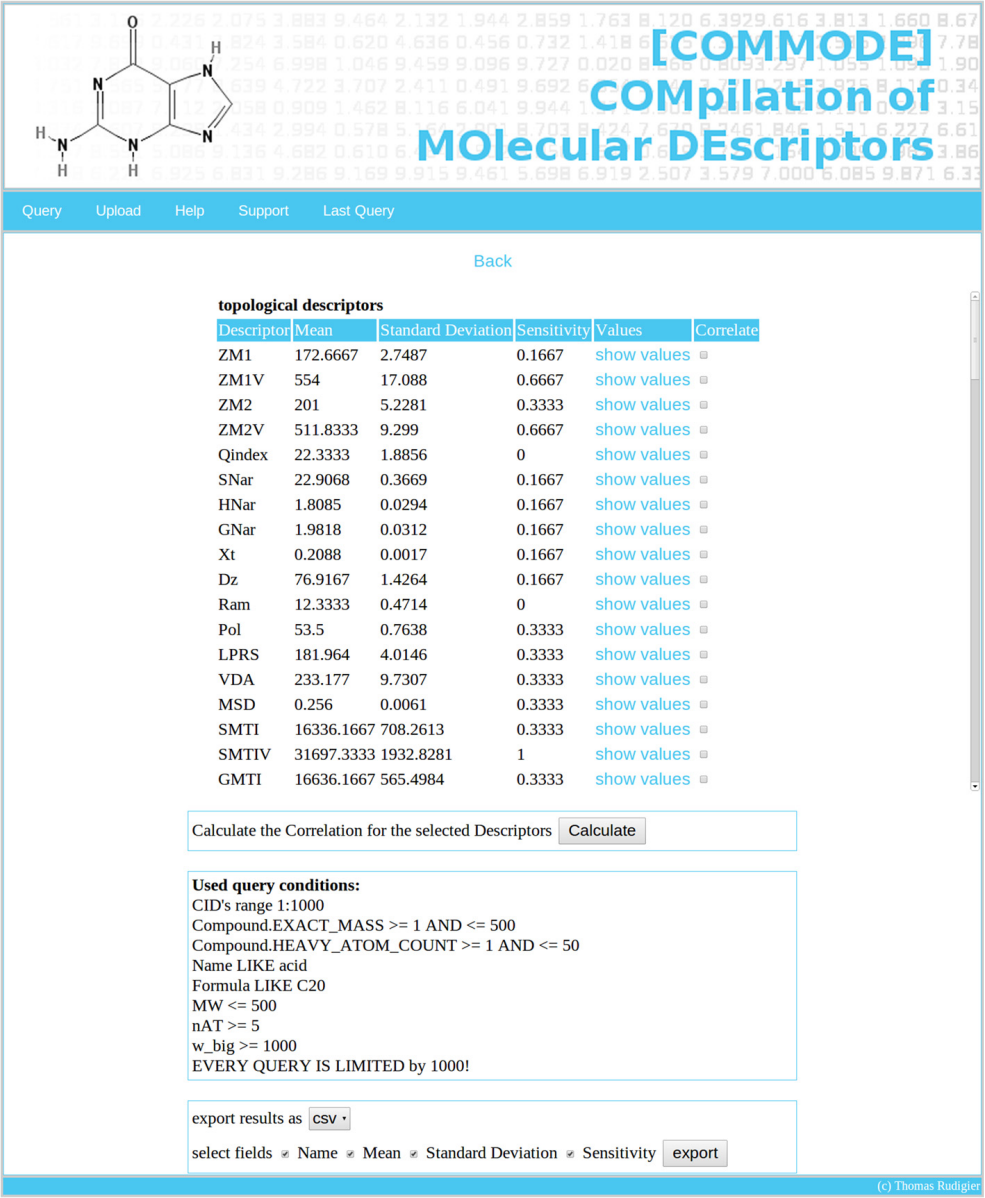
**Screen-shot of COMMODE showing the statistics page.** This screen-shot shows descriptive statistical values for all topological descriptors of compounds from the query shown in Figure 3. Here each line represents one molecular descriptor with the corresponding mean and standard deviation and the discrimination power (sensitivity) [35,36], all calculated for the queried set of compounds. The ’show values’ links can be used to show the values of the given molecular descriptor for all queried compounds, whereas the checkboxes can be used to calculate a correlation matrix between the selected molecules (for the queried compounds).

The same reason described above applies to the analysis of the discrimination power of molecular descriptors. Figure 4 depicts a screen-shot showing molecular descriptors with observed statistical values for the given query. COMMODE is able to estimate Pearson’s correlation coefficient. The analysis of the discriminating power of molecular descriptors has not yet been tackled by using large data sets; except for Hu et al. [33], who investigated the discrimination power of over 4 million structures by calculating EAID numbers. To our knowledge, similar studies have been performed only applying pure structural descriptors by putting the emphasis on rather small sets of compounds [34-36]. Hence, the database system also provides a statistical data analysis part to explore this problem more thoroughly by using a large number of compounds and descriptors.

### Data export

The usage of results within other applications is necessary for scientists, as a lot of downstream analysis can be performed on the integrated data. Therefore, the application supports the following file-formats to export: SDF, SMILES, CSV, MS-Excel^®;^ and XML format. The connection table of the SDF-file is converted from the stored SMILES code using the Chemistry Development Kit (CDK) [37] and opencsv [38] in a specific Java routine. The exported files can further be used in QSPR and QSAR models.

## Conclusions

This work introduces a large chemical database containing chemical compound data and their corresponding molecular descriptor values. These molecular descriptors can be used in QSPR and QSAR models to predict different chemical parameters using the structure of the compounds, and are utilized in drug design.

The published database, COMMODE, includes more than 25 million compounds and about 2,500 computed descriptors. Clearly, COMMODE extends MOLEdb as this database contains only 1,124 molecular descriptors and 234,773 molecules [39,40]. To use our database in QSPR or QSAR models, compounds of interests can be queried either by using different search attributes or by providing a list of PubChem identifiers. Afterwards, results for molecular descriptors can be exported in different file-formats. These results can further be combined with investigated attributes or properties of the given compounds. New models can be designed using these combinations, which can further be used to predict these attributes and properties for other compounds.

As not all molecular descriptors are necessary for the downstream analysis the introduced application is able to calculate descriptive values for each molecular descriptor representing the discrimination power or the correlation coefficient between chosen descriptors.

An additional research area supported by COMMODE is the field of chemical graph theory [7]. COMMODE can be used to analyze the chemical meaning of molecular descriptors [41]. Therefore, descriptive analysis of all descriptors can be performed for all integrated compounds as well as on a particular subset. Also the degeneracy [36,42,43] of all computed and integrated descriptors can be analyzed on different sets of compounds.

Overall, this novel database provides a flexible access to compounds and their related molecular descriptors, which can be used in different research areas.

## Availability and requirements

• **Project name:** COMMODE (COMpilation of MOlecular DEscriptors)

• **Project home page:**http://commode.i-med.ac.at

• **Operating system(s):** Platform independent

• **Programming language:** Java, php

• **Other requirements:** Web Browser

• **Any restrictions to use by non-academics:** none

## Competing interests

The authors declare that they have no competing interests.

## Authors’ contributions

The conceptual idea for COMMODE by using molecular descriptors goes back to MD. Calculation of all molecular descriptors using DRAGON was performed by MD. Furthermore, MD, AD and FES wrote the manuscript. AD and LM performed the implementation of the database. AD and SP implemented Java routines. RG and AG reviewed the database scheme and performed tests. In summary, the project was initiated and coordinated by MD. All authors contributed to the interpreation of the results and read and approved the final manuscript.
